# Identification of low-dose radiation-induced exosomal circ-METRN and miR-4709-3p/GRB14/PDGFRα pathway as a key regulatory mechanism in Glioblastoma progression and radioresistance: Functional validation and clinical theranostic significance

**DOI:** 10.7150/ijbs.57168

**Published:** 2021-03-02

**Authors:** Xinxin Wang, Qinchen Cao, Yonggang Shi, Xiaolong Wu, Yin Mi, Ke Liu, Quancheng Kan, Ruitai Fan, Zhangsuo Liu, Mingzhi Zhang

**Affiliations:** 1 Department of Neurology, The First Affiliated Hospital of Zhengzhou University, Zhengzhou 450052, People's Republic of China.; 2 Department of Radiation Therapy, The First Affiliated Hospital of Zhengzhou University, Zhengzhou 450052, People's Republic of China.; 3 Department of Medical Oncology, The First Affiliated Hospital of Zhengzhou University, Zhengzhou 450052, People's Republic of China.; 4 Department of Pharmacy and Internal Medicine, The First Affiliated Hospital of Zhengzhou University, Zhengzhou 450052, People's Republic of China.; 5 Department of Radiation Oncology, The First Affiliated Hospital of Zhengzhou University, Zhengzhou 450052, People's Republic of China.

**Keywords:** glioblastoma, low-dose radiation, exosome, circ-METRN, progression

## Abstract

Glioblastoma is a central nervous malignancy with a very poor prognosis. This study attempted to explore the role of exosomes induced by low-dose radiation-induced (ldrEXOs) and ldrEXOs-derived circ-METRN in glioblastoma progression and radioresistance at the molecular, cellular, animal, and clinical levels. Results in the present study revealed that low-dose radiation stimulated the secretion of ldrEXOs which delivered high levels of circ-METRN. And circ-METRN-abundant ldrEXOs increased the expression of γ-H2AX, indicating an efficient DNA damage-repair process in glioblastoma cells. The ldrEXOs-derived circ-METRN enhanced the glioblastoma progression and radioresistance via miR-4709-3p/GRB14/PDGFRα pathway. Up-regulating PDGFRα can rescue the tumor-promoting function of ldrEXOs in groups previously treated with inhibition of GRB14. Additionally, in-vivo experiments revealed that treatments with ldrEXOs promoted the growth of xenografted tumors and shortened the survival period. Furthermore, clinical researches indicated that circ-METRN may be transported into the bloodstream by exosomes in the early stages of fractionated radiotherapy. It has important clinical values to detect the serum exosomal circ-METRN in the early stage of radiotherapy, which is not only conducive to predict radioresistance and prognosis but also to assist MRI diagnosis in detecting the very early recurrence of glioblastoma. In summary, this study reveals for the first time that low-dose radiation-induced exosomal circ-METRN plays an oncogenic role in glioblastoma progression and radioresistance through miR-4709-3p/GRB14/PDGFRα pathway, providing mechanistic insights into the roles of circRNAs and a valuable marker for therapeutic targets in glioblastoma.

## Introduction

Glioblastoma is a malignant tumor of the central nervous system with a very high mortality rate 1. The median survival time is less than 2 years 1. Many clinical trials have been conducted to change therapy resistance and improve the survival of glioblastoma patients 2-5. For instance, a convenient approach known as hypofractionated radiotherapy allows the total radiation dose to be converted into larger doses and fewer fractions, and then to be administered in a shorter period, although it was not documented to significantly improve overall survival of glioblastoma patients 5. Despite major advances in radiation technology, the overall outcome of radiotherapy in glioblastoma remains far from optimal, as tumors were identified to be inherently radioresistant and to develop increased radioresistance, especially upon recurrence 2-4. The shorter survival period and the biological resistance to radiotherapy make the role of radiotherapy divergent in glioblastoma 2, 3. It is, thus, essential to select glioblastoma patients who respond well to postoperative chemoradiotherapy and to explore individualized treatment protocols including optimal timing, fractionation, dose, and radiotherapy techniques. Especially for MGMT-unmethylated patients, it is very urgent to find effective indicators and valuable biomarkers to guide more aggressive and effective individualized multimodality therapies.

More importantly, many patients with glioblastoma may suffer undetectable recurrence in the early stages of radiotherapy 4, 6, 7. This very early recurrence is manifested on magnetic resonance imaging (MRI) as a slight abnormal signal, which is difficult to distinguish from cerebral edema, postoperative inflammation, and pseudoprogression 4, 6. Many studies have deeply analyzed the causes of recurrence and found that very early recurrence, insufficient radiotherapy, or interruption of radiotherapy may instead accelerate the progression and promote radioresistance of glioblastoma cells 2-5. Meanwhile, basic laboratory studies have also confirmed that high-grade gliomas are very insensitive to low-dose radiotherapy, and radioresistance is very common in glioblastoma 8-10. Berg et al. demonstrated that all cell lines, including high-grade astrocytoma U-251MG cell lines, developed radioresistance within 2-3 weeks during fractionated radiotherapy 7. Unexpectedly, research on other malignant tumors found that low-dose radiation can promote tumor progression 11, 12.

These earlier studies prompted us to further identify the molecular expression patterns in glioblastoma treated with different radiation doses. Many factors, including bystander effects, exosomes and circular RNAs (circRNAs), serve important roles in the progression, radioresistance, and recurrence of glioblastoma 13, 14. Thereinto, exosomes are nano-sized membrane vesicles with diameters of 30 nm to 100 nm 15. It has previously been documented that exosomes exert a key role in regulating cancer cell-cell communication, tumor-stromal interactions, activation of signaling pathways, and immunomodulation through effectively delivering pathogenic components, such as proteins, RNAs, DNA fragments, and lipids 15. In malignant tumors such as pancreatic cancer, liver cancer, stomach cancer, colon cancer, breast cancer and prostate cancer, tumor cells are very active in secreting exosomes 16-21. As an effective carrier, exosomes can transport various molecular components 16-21. This in turn affects the progression and therapeutic resistance of tumor cells themselves or adjacent cells 16-21. However, few studies identified the functions of exosomes in glioblastoma progression and radiotherapy 22, 23. Emerging data suggest that different doses of radiation can not only promote the production and transport of exosomes but also enhance the absorption of exosomes by other cells 24-26. Radiation-induced exosomes increase the tumor burden, reduce survival**,** induce bystander effects, and promote radiotherapy resistance 15. This allows exosomes to be used as biomarkers to monitor the effects of radiotherapy and predict the prognosis of glioblastoma patients 15. However, the content and component carried by low-dose radiation-induced exosomes (ldrEXOs) remain unclear. There are still some controversies about the regulatory effect of exosomes on radiotherapy resistance and its prognostic value 27, 28.

Early studies indicated that circRNAs played regulatory roles mainly in cells after production 14. Few studies have shown that circRNAs can be transported out of the cell to affect the cellular microenvironment, radioresistance, and bystander effect. A few studies have shown that circRNAs can enhance the radioresistance of malignant tumors 29, but some other studies state that circRNAs can reduce the radioresistance of malignant tumors 16, 29-31. It is obvious that there are still some controversies about the effect pattern of circRNAs on progression and radioresistance of malignant tumor 16, 29-31. Recent studies have revealed that circRNAs are abundant in exosomes 16, 31. Exosomal circRNAs play important roles in the progression of other malignant tumors while the effect of circ-METRN on glioblastoma radiotherapy resistance remains unknown 16, 31.

In this study, we reported for the first time that the identification of circ-MTRN in low-dose radiation-induced exosomes (ldrEXOs), and further explored its functions and underlying mechanisms in glioblastoma progression and radioresistance at molecular, cellular, animal and clinical levels. We identified the role of low-dose radiation-induced exosomal circ-METRN and miR-4709-3p/GRB14/PDGFRα pathway, bringing a novel insight into the investigation of exosomal circRNAs and providing potential targets for anti-glioblastoma therapy.

## Methods

### Cell culture

Human SW1783 and U-118MG glioblastoma cell lines were purchased from the Cell Resource Center of the Shanghai Institute of Biological Sciences. Normal human astrocytes (NHA) were purchased primarily from Sciencell Research Laboratories (Carlsbad, CA, USA) as well. Glioblastoma cells cultured in DMEM medium (Gibco, USA) containing 10% fetal calf serum at 37 °C were placed in a humidified atmosphere containing 5% CO_2_. The cell lines of the same batch were stably subcultured for five generations.

### Exosome isolation and co-culture

To isolate exosomes, glioblastoma cells (SW1783 and U-118MG) previously treated with low-dose radiation (LDR) or high-dose radiation (HDR), were cultured for 48h and the supernatant was collected. To deplete them of the cells and fragments, the supernatants were then centrifuged twice (1,000×g for 10 min and 3,000×g for 30 min at 4 °C). Total Exosome Isolation Reagent (Thermo Fisher Scientific, Inc.) was added overnight, then followed by centrifugation 10,000×g for 1h at 4 °C. Exosomes were resuspended in PBS and stored at a temperature of -80 °C. Exosomes derived from cells in LDR groups (ldrEXO) were added to 10^5^ glioblastoma cells at a concentration of 50 ng/ml serum-free DMEM for 24h. LDR cells were then treated with knockdown of circ-METRN before collection of exosomes (ldrEXO_siCIRC) in the supernatant.

### Transmission electron microscopy

We added the exosome suspension to an equal volume of 4% paraformaldehyde (Nacalai Tesque, Inc., Kyoto, Japan), and applied the mixture to a Formvar/carbon film-coated transmission electron microscope (TEM) grid (Alliance Biosystems, Inc., Osaka, Japan). Subsequently, the sample was fixed by incubation with 1% glutaraldehyde for 5 min, washed with PBS, and incubated with 1% uranyl acetate for 5 min. The sample was then observed using a TEM (Hitachi H7500; Hitachi, Ltd., Tokyo, Japan).

### Nanoparticle tracking analysis

Nanoparticle tracking analysis (NTA) (NanoSight NS300, Malvern Instruments, UK) was applied for size distribution and concentration measurements of exosomes in liquid suspension from the properties of both light scattering and Brownian motion. To detect nanovesicles, the NanoSight NS300 with a 405 nm laser instrument was used. For each sample, five video times of 60 seconds were taken. Data were analyzed using the NTA 3.0 software, and the Hydrodynamic diameters of each particle were calculated using the Stokes-Einstein equation as following: D = kT / (6πηr). [D is the diffusion coefficient, k is Boltzmann's constant; T is the absolute temperature; r is the radius of the particle; η is the viscosity of the fluid, which means a spherical particle moving with the uniform velocity in a continuous fluid].

### Microarray analysis and bioinformatics analysis

CircRNAs were enriched by removing linear RNAs with Rnase R (Epicentre, Madison, WI, USA). Arraystar Human circRNA Array and LncPath Human Cancer Array were used for hybridization, and then the Agilent Scanner G2505C (Jamul, CA, USA) was used for scanning. Heat maps were developed using HemI1.0.1 software (http://hemi.biocuckoo.org/down.php). And then, the downstream signaling pathways of circ-METRN were also analyzed.

### Fluorescence *in situ* hybridization (FISH)

Specific probes to the sequences of circ-METRN (green-labeled, Biosense, Guangzhou, China) (5'- GGCATGGGAGATAGGGAGAC -3') and miR-4709-3p probe (red-labeled, Exiqon, Copenhagen, Denmark) were used *in situ* hybridization. Nuclei were counted by staining with 4, 6-diamidino-2-phenylindole (DAPI). All procedures were conducted according to the manufacturer's instructions (Genepharma, Shanghai, China). All images were acquired on a Zeiss LSM880 NLO (2+1 with BIG) confocal microscope system (Leica Microsystems, Mannheim, Germany).

### Quantitative real-time PCR (RT-PCR) and RNase R treatment

RT-PCR was performed as previously described 14. The primers used for RT-PCR can be found in **Supplementary Material.**

Transcription was prevented by the addition of 2 mg/mL Actinomycin D or DMSO (Sigma-Aldrich, St. Louis, MO, USA) as a negative control (NC). Total RNA (2 μg) was incubated for 30 min at 37 °C with 3 U/μg of RNase R (Epicentre, Madison, WI, USA). After treatment with RNase R, the RNA expression levels of linear-METRN and circ-METRN were detected by RT-PCR.

### RNA interference and transfection assays

Small interfering RNAs (siRNAs) targeting linear transcript or targeting the backsplice sequence of circRNA were designed and synthesized by RiboBio (Guangzhou, China). RNA interference and transfection assays were performed as previously described 14. More details about the siRNAs used in this study can be found in **Supplementary Material.**

### MTT assays and cell apoptosis assays and colony formation assay

At the indicated time point after transfection, cells were collected by centrifugation after incubation with 5.0 mg/ml 3-(4,5-dimethyl-2-thiazolyl)-2,5-diphenyl-2-H-tetrazolium bromide (MTT) Dimethyl sulfoxide (200 μl) was added into the sediments, and then spectrophotometry at 490 nm was used for the measurement of the absorbance. To identify cell apoptosis, cells were also stained using an annexin V apoptosis kit (eBiosciences, USA) and analyzed using flow cytometry.

For colony formation capacity detection, glioblastoma cells were plated in six-well plates in triplicate (400 cells/well). The cells were incubated for 2 weeks, and the culture media was refreshed per 3d. The colonies were stained with crystal violet. The colonies including 50 cells or more cells were counted.

### Wound healing assays and transwell assays

Wound healing assays and transwell assays were performed as previously described 14. Transwell inserts (Corning, NY, USA) and Matrigel (BD Biosciences, CA, USA) were purchased. An inverted microscope (Motic Instruments, Richmond, BC, Canada) was applied in capturing images.

### Luciferase reporter assays

Human embryonic kidney (HEK) 293T cell lines were purchased from the Cell Resource Center of the Shanghai Institute of Biological Sciences. Luciferase reporter assays were performed as previously described 14.

### Western blot analysis

Western blot was performed as previously described 14. More details about antibodies can be found in **Supplementary Material.**

### Orthotopic xenograft model

SW1783 and U-118MG cells were injected (5×10^7^ cells/ml) into the right striatum through a burr hole in the skull of BALB/C nude mice (4-6 weeks old, 18-22 g, six female mice per group) using a 10-μl Hamilton syringe. Before inoculation, mice were anesthetized with an intraperitoneal injection of 0.1% pentobarbital (0.1 ml) in an animal stereotaxic apparatus. After inoculation, the wound was sutured to avoid infection. Tumor growth was monitored and measured via bioluminescence imaging *in vivo* by the injection of 10 μl/g bodyweight of D-luciferin using the IVIS Spectrum system (Perkin Elmer, Waltham, USA) according to the recommended procedure. Animals were daily monitored for cachexia (evaluated by bodyweight waste). Animals that lost about 20% of the bodyweight were euthanized. All animals' experimental protocols were approved by the Ethics Committee of Zhengzhou University and the Experimental Animal Center of Zhengzhou University for compliance with the National Institutes of Health for use of laboratory animals or equivalent. All methods were carried out following the relevant guidelines and regulations. Every effort was made to minimize the number of animals used and their suffering.

### Irradiation procedure

Exponentially growing cells were seeded in culture flasks, 72 h before the first experimental irradiation, and subsequently exposed to a variety of single doses: 2 Gy (approximately the daily dose used in humans), 4 Gy, 6 Gy, 8 Gy, and 10 Gy of the X-ray beam in a linear accelerator (Elekta Synergy, Sweden). Six of the seeded plates served as controls and received no irradiation. The medium was refreshed twice every week. Cell counts were performed using a Coulter Counter (Beck-man Coulter, Inc., Fullerton, USA).

Mice were locally irradiated at a single dose of 10Gy under anesthesia. Control animals were anesthetized and sham irradiated. Mice that presented neurological symptoms (i.e. hydrocephalus, seizures, inactivity, and/or ataxia) or moribund were also sacrificed.

### γ-H2AX staining

Cells derived from glioblastoma biopsy specimens, treated with ldrEXO or ldrEXO_siCIRC were exposed to a radiation dose of 10Gy. Six hours or 24 h post-radiation, cells were fixed, and intracellular staining with an Alexa Fluor 488 anti-H2AX-Phosphorylated (Ser139) antibody was performed according to the manufacturer's protocol (BioLegend). Stained cells were analyzed by the BD Accuri C6 cytometer and the data were analyzed by the BD Accuri C6 software.

### Clinical research and human specimens

From January 1, 2015 to December 31, 2019, at the First Affiliated Hospital of Zhengzhou University, glioblastoma patients who underwent surgical resections were reviewed. The pathological classification was based on the WHO 2007 classification of tumors by two experienced neuropathologists. The inclusion and exclusion criteria were shown in **Supplementary Material.** Disease-free survival (DFS) was calculated from the date of complete resection to any recurrent disease, distant metastasis, or death. Overall survival (OS) was calculated from the date of surgery to the date of death from any cause.

Fresh frozen pathological specimens and serum specimens were both obtained. Serum specimens were acquired from patients before operation, before radiotherapy, during radiotherapy (in the first week of radiotherapy), or after radiotherapy, respectively. The circ-METRN levels in glioblastoma tissues and serum exosomes were analyzed and compared. To compare the molecular expression differences among gliomas of different grades (n = 12, each group), some other specimens, including peritumoral normal brain tissues (PNBTs) and Grade I-III gliomas, were also collected. PNBTs were used as NCs. All experimental protocols in this section were approved by the Ethics Committee of Zhengzhou University. Written informed consent was signed and obtained from all individual participants or their legal guardians in this study.

### Statistical analysis

All statistical tests were two-sided. Experimental data are shown as the mean ± standard deviation (SD). Error bars were obtained according to the standard deviation. Student's two-tailed unpaired t test was used to determine the statistical significance. And a P value < 0.05 was considered statistically significant. The Kaplan-Meier method and log-rank test were used to compare survival rates. The multivariate survival analysis was applied to identify independent prognostic factors. Statistical analyses were performed using SPSS software version 17.0 (SPSS Inc., Chicago, IL, USA) and R 2.8.0 statistical package (the R Core Team, Vienna, Austria).

## Results

### LDR stimulated the secretion of glioblastoma exosomes that are enriched with high levels of circ-METRN, and circ-METRN-abundant ldrEXOs may modulate DNA damage response

**Figure 1:**
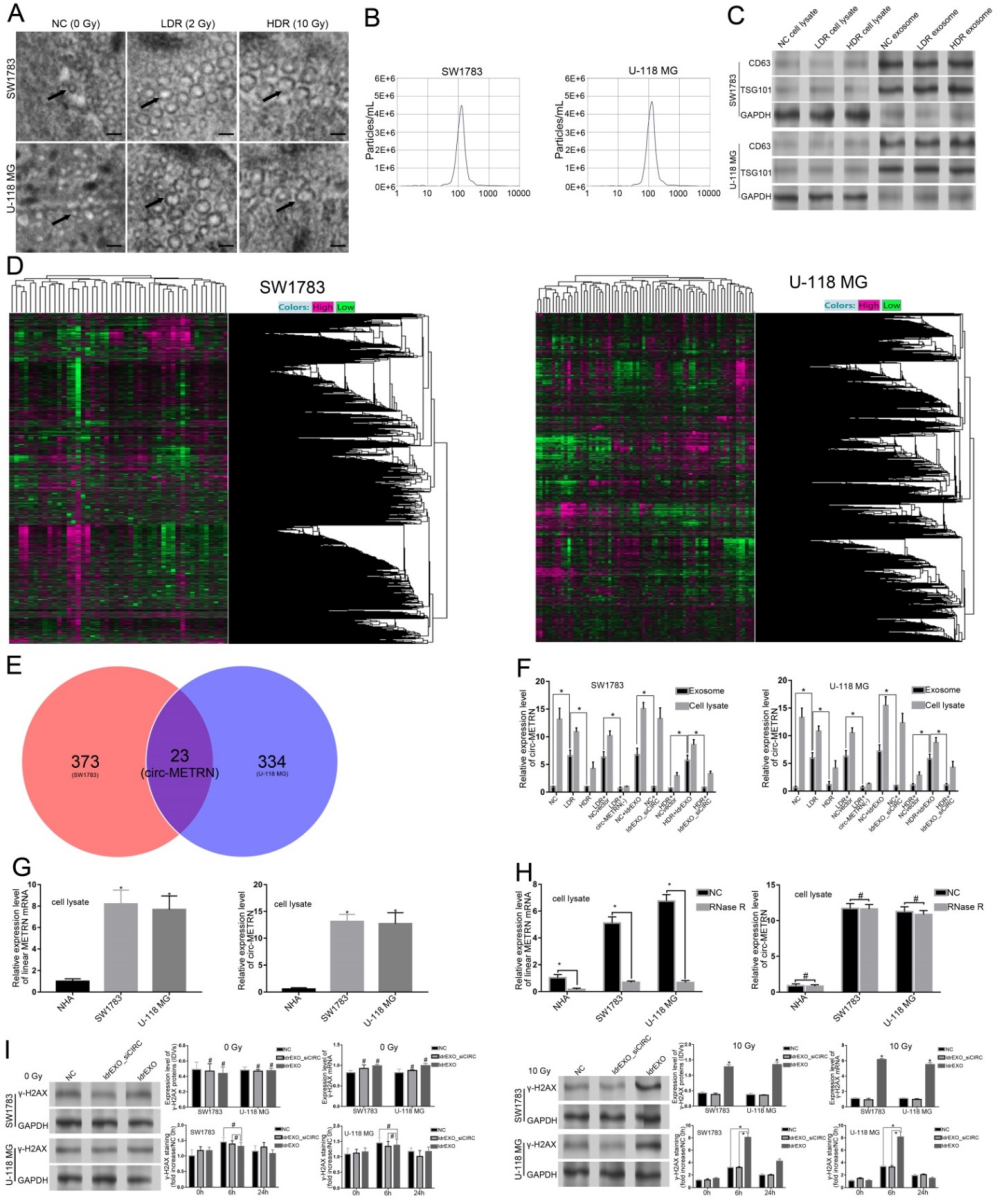
** Characterization of exosomes derived from glioblastoma cell lines treated with low-dose radiation. A.** Electron microscope scanning of exosomes (arrows) isolated from SW1783 and U-118 MG cell lines (n=6) (scale bar: 50 nm). **B.** The size range of the low-dose radiation-induced exosomes (ldrEXOs) isolated from SW1783 and U-118 MG cell lines checked by NAT analysis. **C.** Western blot analysis of exosomal markers, including CD63 and TSG101 (n=6). **D.** Heat maps of the expression profile consisting of three pairs of exosomes in SW1783 and U-118 MG cells treated with low-dose radiation (LDR) and control cells (NC) by RNA sequencing (23 pairs). **E.** A Venn diagram of the intersection between results of the differential expression of SW1783 and U-118 MG cells. **F.** RT-PCR analyses of the content of exosomal circ-METRN in different groups (n=6). **G.** The relative expression levels of circ-METRN and its cognate linear mRNA in normal human astrocytes (NHA) and glioblastoma cell lines (n=6). **H.** Expression levels of circ-METRN and its cognate linear mRNA in glioblastoma cell lines after treatment with Rnase R. **I.** ldrEXOs increased the expression of γ-H2AX, indicating an efficient DNA damage-repair process in glioblastoma cells (n=6). ldrEXOs with inhibition of circ-METRN (ldrEXOs_siCIRC) did not significantly exhibit the promoting effect, indicating the important role of exosomal circ-METRN in the DNA damage-repair process in glioblastoma cells (n=6). *P<0.05, #*P*>0.05.

To identify and analyze exosomes induced by different doses of irradiation, electron microscope scanning and NAT analysis were both performed (Figure 1A-B). Expressions of CD63 and TSG101 proteins were also detected by Western blot (Figure 1C). After microarray analysis for ldrEXOs, a total of 23 circRNAs, including circ-METRN, were identified as abnormally expressed in glioblastoma exosomes (Figure 1D-E). The expression of exosomal circ-METRN in the LDR group was significantly higher than that in the NC group and HDR group (Figure 1F). In groups treated with ldrEXOs, levels of circ-METRN in exosomes and cell lysates were both increased (Figure 1F). The relative expression levels of circ-METRN and its cognate linear mRNA before and after treatment with Rnase R were also analyzed in NHA and glioblastoma cell lysates (Figure 1G-H).

Ionizing radiation is known to induce DNA damages leading to lethal cytotoxicity, and high activation of the DNA damage repair is an important component of radioresistance of glioblastoma in particular. To determine whether inhibition of exosomal circ-METRN affected radiation-induced phosphorylation of H2AX (γ-H2AX), a marker of DNA breaks, we further examined the expression of γ-H2AX. As expected, γ-H2AX staining increased rapidly following radiation and returned to basal level at 24 h in cells treated with ldrEXOs (Figure 1I), indicating an efficient DNA damage-repair process in glioblastoma cells. However, ldrEXOs with inhibition of circ-METRN (ldrEXOs_siCIRC) did not significantly exhibit the promoting effect, suggesting the important role of exosomal circ-METRN in the DNA damage-repair process in glioblastoma cells.

### Circ-METRN-abundant ldrEXOs promoted progression and radioresistance of glioblastoma cell lines

**Figure 2:**
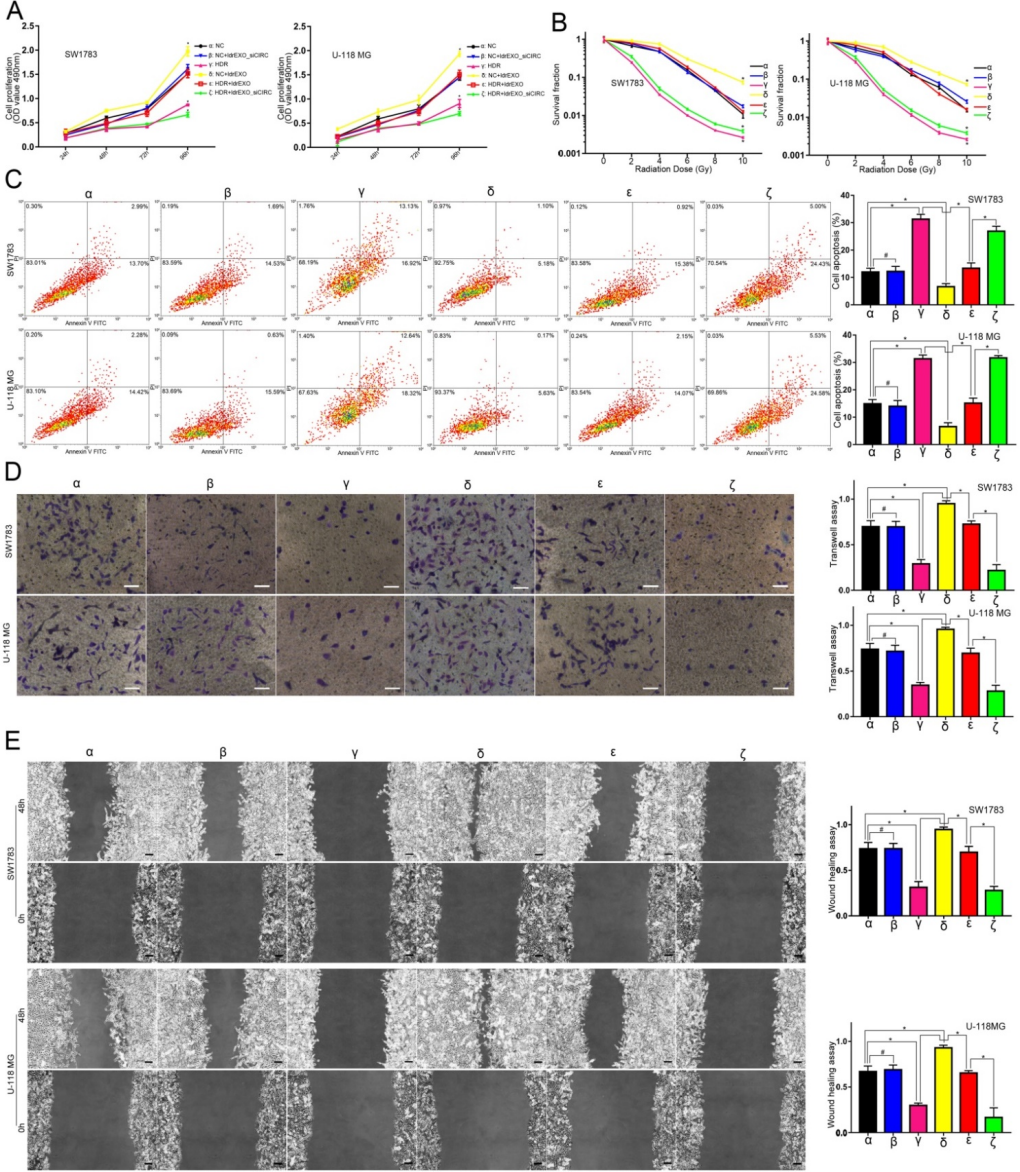
** circ-METRN-abundant ldrEXOs promote progression and radioresistance of glioblastoma cell lines. A.** Cell proliferation assays were conducted to analyze the effects of circ-METRN-abundant ldrEXOs on glioblastoma cell proliferation ability (**δ**, **ε**). ldrEXOs_siCIRC: ldrEXOs with inhibition of circ-METRN (**β**, **ζ**). **B.** Radiation sensitivity was tested with colony formation assays in both cell lines. **C.** Apoptosis resistance was assessed by flow cytometry with annexin V-FITC/propidium iodide (PI). **D**-**E.** Transwell assays and wound healing assays were used to analyze glioblastoma cell migration and invasion ability, respectively. *P<0.05, #*P*>0.05.

Compared with the NC group (**α**), ldrEXOs with high levels of circ-METRN (**δ, ε**) promoted proliferation, radioresistance, apoptosis resistance, invasion, and migration abilities of glioblastoma cell lines while ldrEXOs with inhibition of circ-METRN (ldrEXOs_siCIRC) (**β, ζ**) did not significantly exhibit the promoting effect (Figure 2A-E). ldrEXOs with high levels of circ-METRN, instead of ldrEXOs_siCIRC (**ζ**), also rescued proliferation, radioresistance, apoptosis resistance, invasion, and migration abilities of glioblastoma cell lines previously treated with high-dose radiation (HDR) (**ε**) (Figure 2A-E).

### Exosomal circ-METRN was efficiently transported into glioblastoma cells by ldrEXOs and acts as a miR-4709-3p sponge

**Figure 3:**
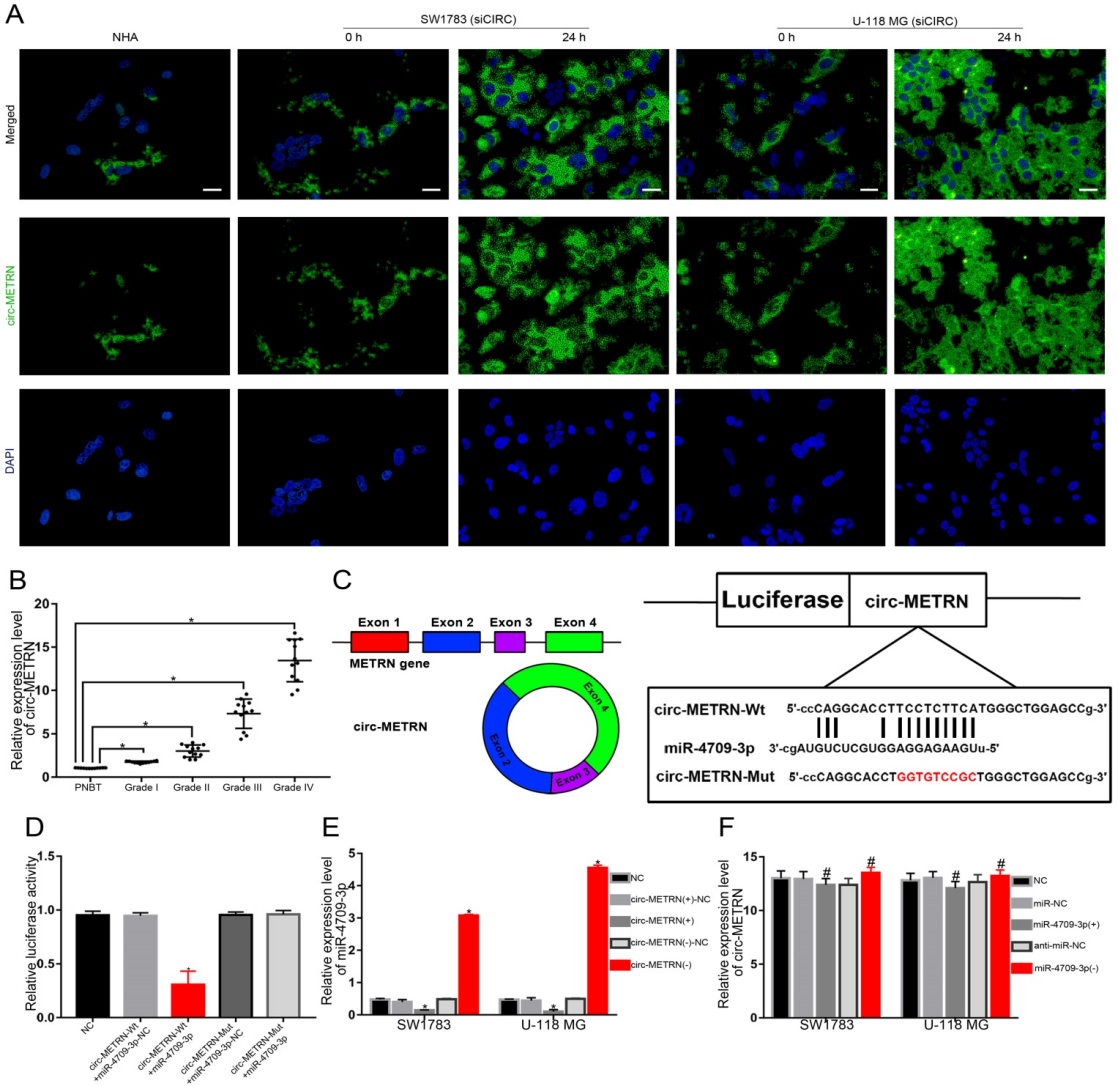
** Exosomal circ-METRN was efficiently transported into glioblastoma cells by ldrEXOs and acts as a miR-4709-3p sponge. A.** After treatments with ldrEXOs, the levels of circ-METRN in circ-METRN-knockdown (siCIRC) glioblastoma cells were significantly improved (n = 6) (scale bar: 20 µm). **B.** Expression levels of circ-METRN in PNBT and glioblastoma (n = 12). **C.** The cartoon of circ-METRN arose from the METRN gene and the predicted miR-4709-3p binding sites in circ-METRN (Wt) and the designed mutant sequences (Mut). **D.** Luciferase reporter assay of HEK 293T cells with different co-transfection treatments. **E.** RT-PCR analysis showed that circ-METRN regulated miR-4709-3p expression in the circ-METRN(+) group and the circ-METRN(-) group, respectively. **F.** RT-PCR analysis further verified that miR-4709-3p did not significantly attenuate circ-METRN expression either in the miR-4709-3p(+) group or in the miR-4709-3p(-) group. *P<0.05, #*P*>0.05.

To verify whether circ-METRN can be effectively transported by ldrEXOs among glioblastoma cells, circ-METRN-knockdown (siCIRC) cells of glioblastoma were treated with ldrEXOs. As a result, the level of circ-METRN in these cells was significantly improved (Figure 3A). Then, the expression level of circ-METRN in different grades of glioma tissues was proved to be very different (Figure 3B). What's more, circ-METRN was predicted to share miRNA response elements (MREs) of miR-4709-3p (Figure 3C). Further luciferase reporter assays confirmed that circ-METRN had a sponging effect on miR-4709-3p (Figure 3D). Circ-METRN can affect the expression levels of miR-4709-3p (Figure 3E) while miR-4709-3p did not impact the expression of circ-METRN (Figure 3F).

### The miR-4709-3p expression was significantly affected by the ldrEXO-transported circ-METRN and GRB14 is a direct target of miR-4709-3p

**Figure 4:**
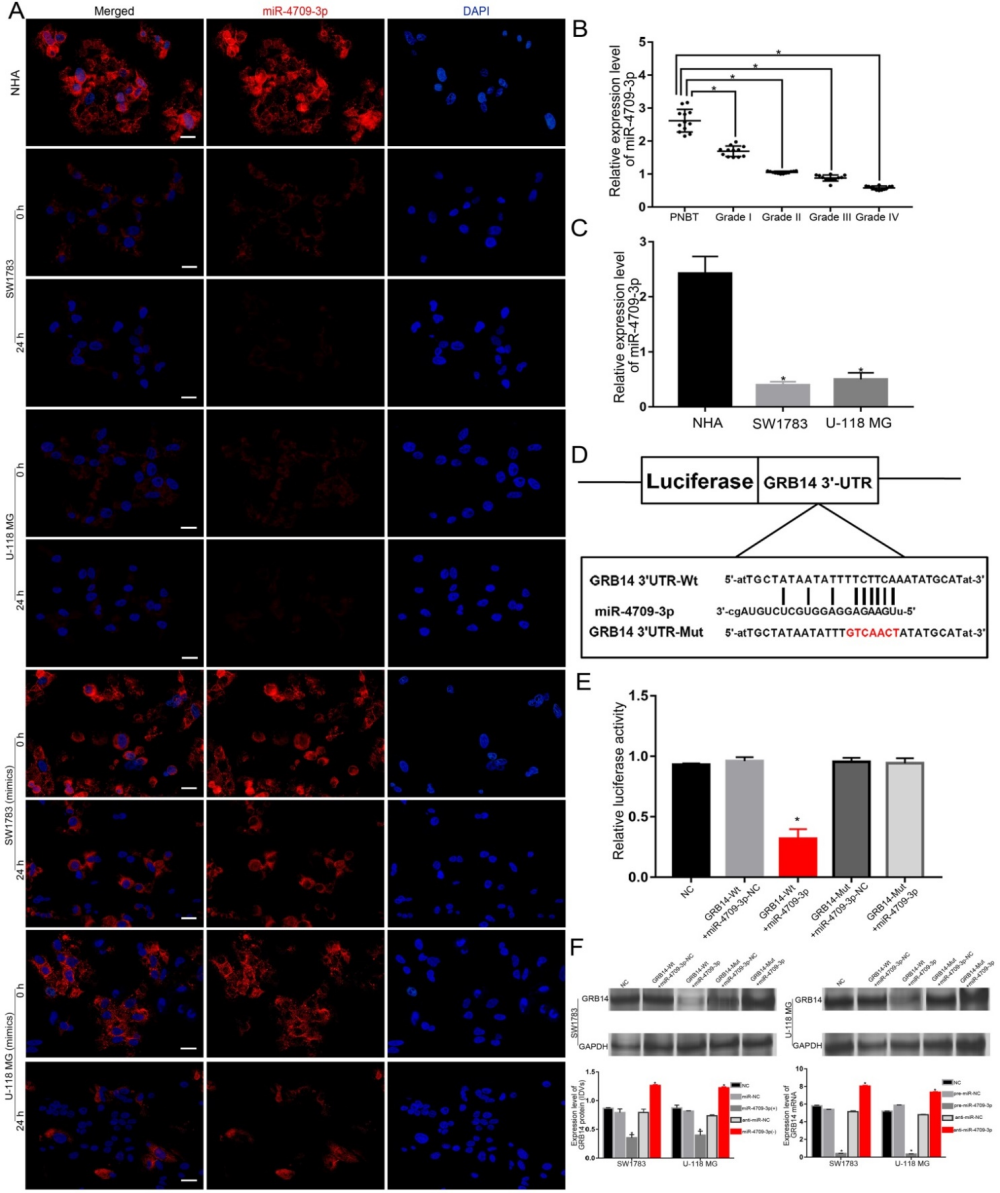
** miR-4709-3p was significantly affected by the ldrEXO-transported circ-METRN and exerts its regulatory functions by targeting GRB14. A.** After treatments with ldrEXOs, the levels of miR-4709-3p were significantly impacted in glioblastoma cells with or without the treatment of miR-4709-3p mimics (n = 6) (scale bar: 20 µm). **B.** Expression levels of miR-4709-3p in PNBT and glioblastoma (n = 12). **C.** Expression levels of miR-4709-3p in normal human astrocytes (NHA) and glioblastoma cells (n = 6). **D.** The predicted miR-4709-3p binding sites in GRB14 (GRB14 3'UTR-Wt) and GRB14 3'UTR-Mut. **E.** Luciferase reporter assay of HEK 293T cells with different co-transfection treatments. **F.** The expression level of GRB14 protein and mRNA was regulated by miR-4709-3p in glioblastoma cells. *P<0.05, #*P*>0.05.

Without the treatment of miR-4709-3p mimics, miR-4709-3p was under-expressed in glioblastoma cells and tissues (Figure 4A-C). Despite the low expression of miR-4709-3p in glioblastoma, the sponging effect of the ldrEXO-transported circ-METRN was still observed (Figure 4A). To verify whether exosomal circ-METRN can still play a sponging role in the high level of miR-4709-3p, we also observed the effect of ldrEXO treatment on the miR-4709-3p level in glioblastoma cells treated with the previous transfection of miR-4709-3p mimics (Figure 4A). Results revealed that the level of miR-4709-3p in these cells was also significantly decreased after treatment with ldrEXOs (Figure 4A). Moreover, GRB14 was then predicted as a direct target of miR-4709-3p (Figure 4D). Further luciferase reporter assays confirmed that miR-4709-3p had a targeting relationship to GRB14 (Figure 4E). And miR-4709-3p can affect the expression levels of GRB14 mRNA and protein (Figure 4F).

### Low-dose radiation-induced exosomal circ-METRN promotes glioblastoma cell progression and radioresistance by regulating the miR-4709-3p/GRB14 axis

**Figure 5:**
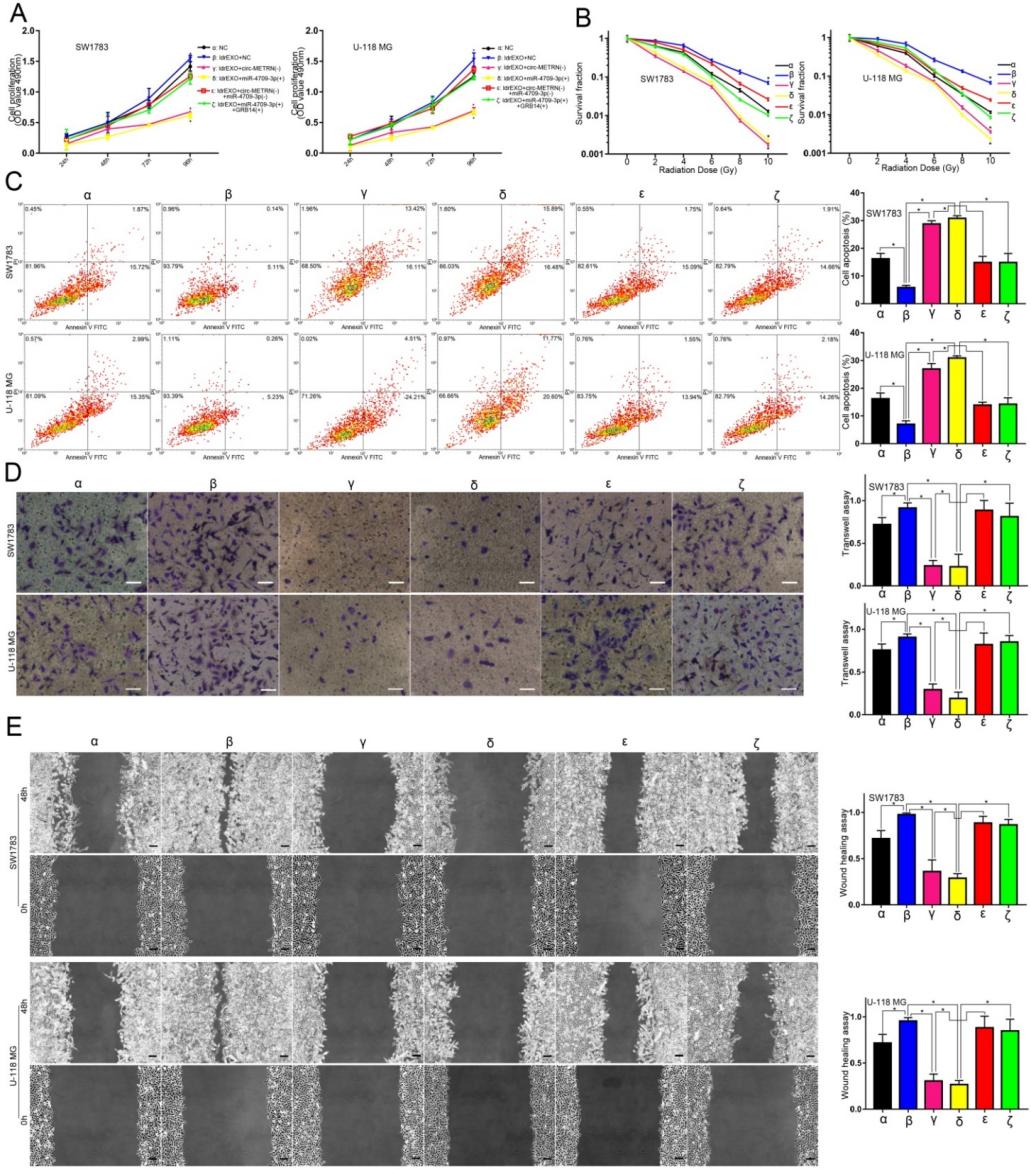
** Low-dose radiation-induced exosomal circ-METRN promotes glioblastoma cell progression and radioresistance by regulating the miR-4709-3p/GRB14 axis. A.** Proliferation-promoting effects of circ-METRN-abundant ldrEXOs on glioblastoma cells can be attenuated by miR-4709-3p (δ) and can be rescued by GRB14 (ζ). **B.** Radioresistance-promoting effects of circ-METRN-abundant ldrEXOs on glioblastoma cells can be attenuated by miR-4709-3p (δ) and can be rescued by GRB14 (ζ). **C.** Apoptosis resistance was assessed by flow cytometry with annexin V-FITC/propidium iodide (PI). **D-E.** Transwell assays and wound healing assays were used to analyze glioblastoma cell migration and invasion ability, respectively. *P<0.05, #*P*>0.05.

ldrEXOs promoted proliferation, radioresistance, apoptosis radioresistance, invasion, and migration abilities of glioblastoma cell lines (**β**) while knockdown of circ-METRN (**γ**) or up-regulation of miR-4709-3p (**δ**) attenuated these abilities of glioblastoma cell lines previously treated with ldrEXOs (Figure 5A-E). Knockdown of miR-4709-3p (**ε**) or up-regulation of GRB14 (**ζ**) rescued progression and radioresistance of glioblastoma cell lines (Figure 5A-E).

### GRB14 has a regulatory function on PDGFRα. And ldrEXO-derived circ-METRN may have a positive effect on downstream pathways in glioblastoma cells

**Figure 6:**
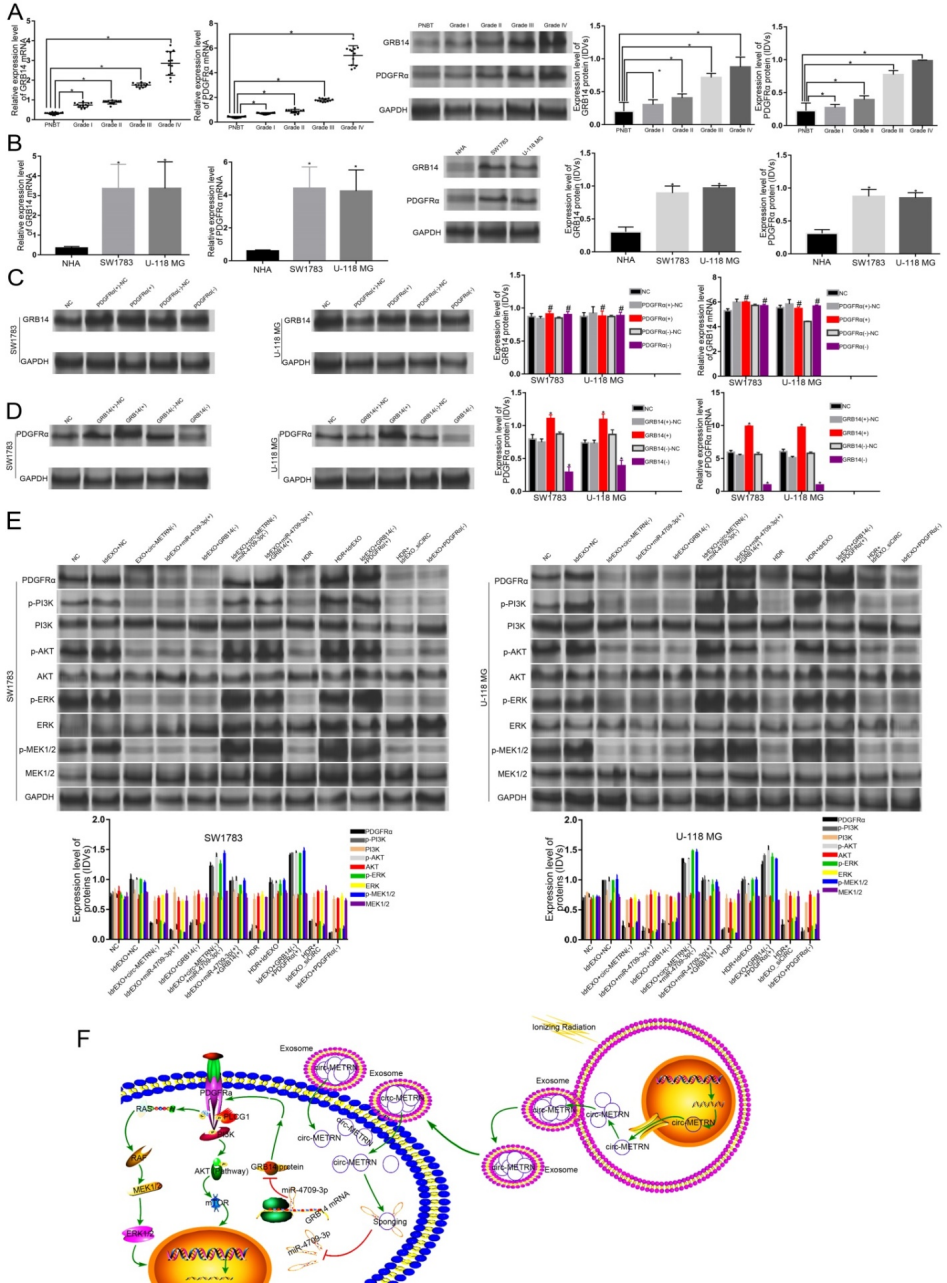
** Impacts of ldrEXOs and ldrEXO-derived circ-METRN on the downstream pathway in glioblastoma cells. A.** Expression levels of GRB14 and PDGFRα in PNBT and glioblastoma tissues (n = 12). **B.** The relative expression levels of GRB14 and PDGFRα in NHA and glioblastoma cell lines. **C.** The expression levels of GRB14 protein and mRNA were not affected by PDGFRα in the PDGFRα(+)/PDGFRα(-) group. **D.** RT-PCR and western-blot analysis further verified that GRB14 can regulate the PDGFRα expression in the GRB14(+)/GRB14(-) group, respectively. **E.** Western blot analysis showed that PDGFRα, p-PI3K, PI3K, p-AKT, AKT, p-ERK, ERK, p-MEK1/2, and MEK1/2 were regulated by ldrEXOs, exosomal circ-METRN, and miR-4709-3p/GRB14 axis in SW1783 and U-118MG cells. **F.** The schematic cartoon of the mechanism of low-dose radiation-induced exosomal circ-METRN via miR-4709-3p/GRB14/PDGFRα pathway in glioblastoma cells was shown. *P<0.05, #*P*>0.05.

GRB14 and PDGFRα exhibited high expression levels in glioblastoma tissues and cell lines (Figure 6A-B). There was no significant difference in the expression levels of GRB14 mRNA and protein after knocking down and/or increasing the expression level of PDGFRα (Figure 6C). However, up-regulating GRB14 increased PDGFRα expression level, and down-regulating GRB14 decreased PDGFRα expression level (Figure 6D).

The impact of ldrEXOs, exosomal circ-METRN, and miR-4709-3p/GRB14 axis on the proteins of PDGFRα, p-PI3K, PI3K, p-AKT, AKT, p-ERK, ERK, p-MEK1/2, and MEK1/2 were also analyzed by western blot (Figure 6E). The mechanism of low-dose radiation-induced exosomal circ-METRN via miR-4709-3p/GRB14/PDGFRα pathway in glioblastoma cells was elucidated by using the schematic cartoon (Figure 6F).

### GRB14 may play glioblastoma-promoting roles through regulating the downstream PDGFRα after treatments with ldrEXOs

**Figure 7:**
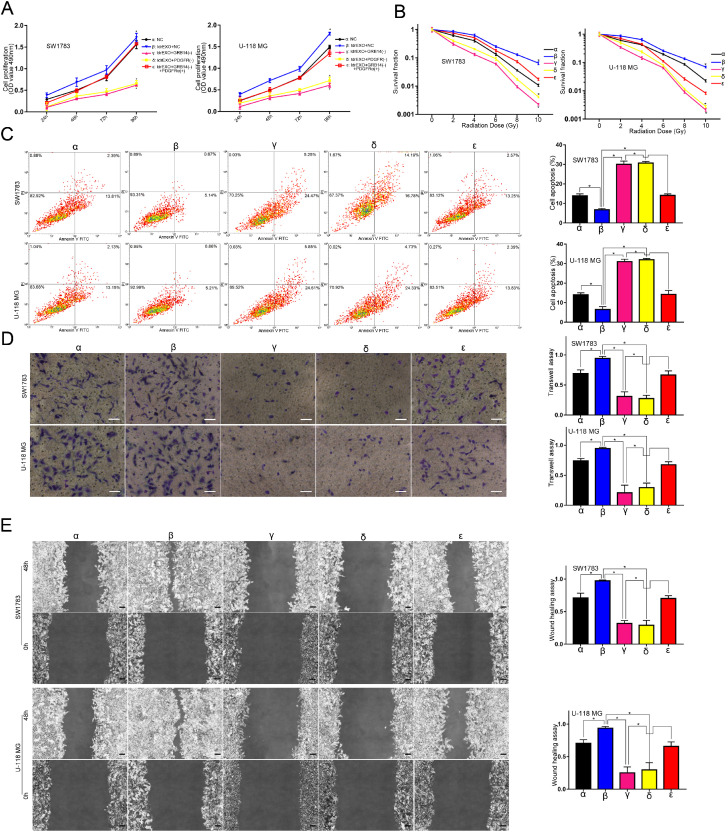
** GRB14 may play glioblastoma-promoting roles through regulating the downstream PDGFRα after treatments with ldrEXOs. A.** Proliferation-promoting effects of circ-METRN-abundant ldrEXOs on glioblastoma cells can be attenuated by GRB14 (γ) inhibition and can be rescued by PDGFRα (ε). **B.** Radioresistance-promoting effects of circ-METRN-abundant ldrEXOs on glioblastoma cells can be attenuated by GRB14 inhibition (γ) and can be rescued by PDGFRα (ε). **C.** Apoptosis resistance was assessed by flow cytometry with annexin V-FITC/propidium iodide (PI). **D-E.** Transwell assays and wound healing assays were used to analyze glioblastoma cell migration and invasion ability, respectively. *P<0.05, #*P*>0.05.

Knockdown of GRB14 (**γ**) or PDGFRα (**δ**) attenuated proliferation, radioresistance, apoptosis radioresistance, invasion, and migration abilities of glioblastoma cell lines previously treated with ldrEXOs (Figure 7A-E). Up-regulation of PDGFRα (**ε**) rescued progression and radioresistance of glioblastoma cell lines previously treated with knockdown of GRB14 (Figure 7A-E).

### Low-dose radiation-induced exosomal circ-METRN promotes glioblastoma progression and radioresistance *in vivo*

**Figure 8:**
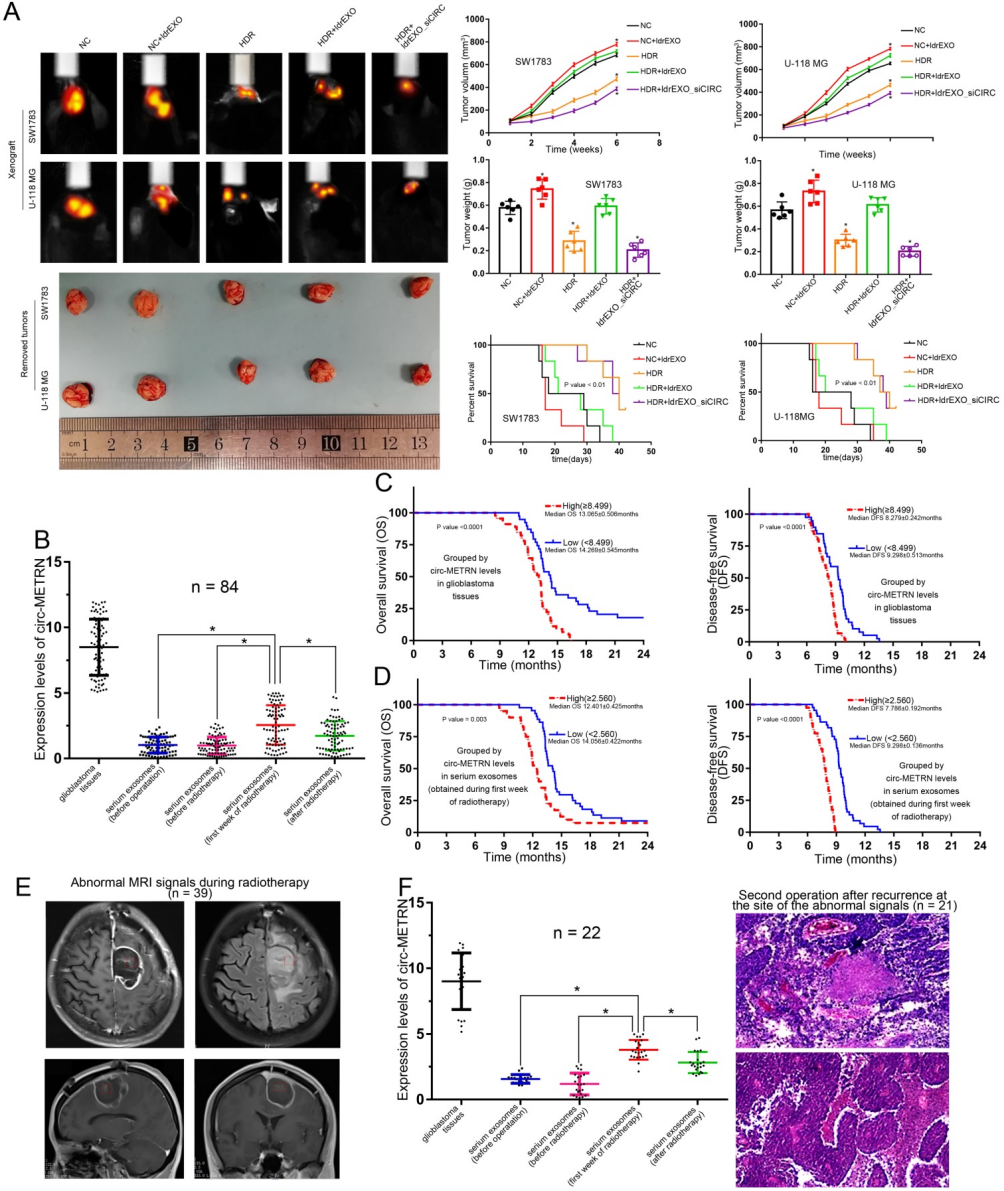
** In-vivo experiments on the role of low-dose radiation-induced exosomal circ-METRN and clinical research on its values in glioblastoma progression and radioresistance. A.** *In vivo* fluorescence images of glioblastoma-bearing mice intravenously receiving SW1783 and U-118MG cells with different treatments were obtained before euthanizing mice. Representative pictures of removed xenograft tumors, as well as tumor volume, tumor weight and survival time, are presented (n=6, each group). **B.** circ-METRN levels in glioblastoma tissues and serum exosomes (obtained before the first operation, before radiotherapy, during the first week of radiotherapy, or after radiotherapy) were detected and compared. **C-D.** Exosomal circ-METRN level detected in the early stage of radiotherapy, as well as circ-METRN level in glioblastoma tissue, was an independent prognostic factor affecting OS and DFS. **E.** MRI reexamination during radiotherapy indicated abnormal signals (red box) in 39 patients. **F.** 21 of these 22 patients with high levels of circ-METRN and abnormal MRI signals during radiotherapy were then identified pathologically as recurrence at the site of abnormal signals. *P<0.05, #*P*>0.05.

The in-vivo experiment showed that treatments with ldrEXOs resulted in the rapid growth of xenografted tumors and a shortened survival period (Figure 8A). HDR treatments reduced tumor growth and prolonged survival period. Moreover, ldrEXOs with high levels of circ-METRN rescued tumor-growth ability of glioblastoma previously treated with HDR while ldrEXOs_siCIRC did not significantly exhibit the rescue effect (Figure 8A).

### Low-dose radiation-induced exosomal circ-METRN may be delivered into the bloodstream in the early stages of fractionated radiotherapy

In this study, detailed information about a total of eighty-four glioblastoma patients were collected (**Table 1**). Further detection revealed that circ-METRN level (Mean±SD) in Glioblastoma tissues was 8.499±0.233 while circ-METRN levels (Mean±SD) in serum exosomes were 1.025±0.067 (before operation), 0.995±0.071 (before radiotherapy), 2.560±0.164 (during the first week of radiotherapy), and 1.737±0.122 (after radiotherapy), respectively. The level of serum exosomal circ-METRN detected in the first week of radiotherapy was significantly higher than that before radiotherapy (Figure 8B). These results suggested that high levels of circ-METRN may be delivered into the bloodstream by exosomes in the early stage of fractionated radiotherapy. Compared with the whole course of radiotherapy, the radiation dose delivered in the surgical area of the tumor bed was not high in the early stage of radiotherapy. Serum exosomal circ-METRN detected in the first week of radiotherapy was considered as low-dose radiation-induced exosomal circ-METRN.

### High levels of serum exosomal circ-METRN can not only predict a poor prognosis but also assist MRI diagnosis in early detection of recurrence in glioblastoma patients

Survival analysis demonstrated that glioblastoma patients exhibited poor prognosis (1-year OS 75.0%, 2-year OS 8.3%; 1-year DFS 2.4%, 2-year DFS 0.0%). And median OS and DFS were 13.313±0.085 months and 8.54±0.191 months, respectively. Multivariate analysis revealed that exosomal circ-METRN (detected in the early stage of radiotherapy) was an independent prognostic factor for OS and DFS (**Supplementary Material: Table S6**). Glioblastoma patients displaying high levels of exosomal circ-METRN exhibited a worse prognosis than other groups (Figure 8C-D). Rapid recurrence after postoperative radiotherapy occurred in patients with high levels of exosomal circ-METRN (detected in the early stage of radiotherapy) (Figure 8D), suggesting significant radiotherapy resistance in these glioblastoma patients. It may be very essential for these patients to seek other more effective and aggressive individualized treatments.

We also explored the diagnostic value of serum exosomal circ-METRN detected in the early stage of radiotherapy. MRI reexamination during radiotherapy indicated abnormal signals in 39 patients (Figure 8E), 22 of whom had high expression of exosomal circ-METRN in the early stage of radiotherapy. And 21 of these 22 patients were subsequently diagnosed by pathology following secondary surgery as recurrence at the site of abnormal signals (Figure 8F). Of the 17 patients with low expression of exosomal circ-METRN in the early stage of radiotherapy, only 3 patients were pathologically diagnosed as having relapses at the abnormal-signal site after secondary surgery. Then, we rediagnosed glioblastoma recurrence using a novel method combining MRI with serum exosomal circ-METRN (detected in the early stage of radiotherapy). According to this combined diagnosis method, cases with both abnormal MR signals and high levels of serum exosomal circ-METRN detected in the early stage of radiotherapy were defined as having very early recurrence (positive); cases with no abnormal MRI signals and with low levels of serum exosomal circ-METRN are not considered to have recurrences (negative) (**Supplementary Material: Table S7**). Meanwhile, based on pathological diagnosis, recurrence on the location of abnormal MRI signals was identified as a positive diagnosis; non-malignant lesion on the location of abnormal MRI signals or recurrence on other locations out of the abnormal-signal area was identified as a negative diagnosis (**Supplementary Material: Table S7**). According to the analysis, the true positive rate (0.875, 21/24), true negative rate (0.960, 24/25), and accuracy (0.918, 45/49) of this combined diagnosis method were significantly higher than those of MRI diagnosis alone [true positive rate 0.167 (4/24), true negative rate 0.08 (2/25) and accuracy 0.122 (6/49)] (**Supplementary Material: Table S7**).

## Discussion

The high mortality rate of glioblastoma is closely related to the recurrence of this malignant tumor after postoperative radiochemotherapy 1. The advancement of radiotherapy technology has neither reduced the recurrence rate of glioblastoma nor has it effectively changed the radioresistance of glioblastoma cells, especially for MGMT-unmethylated patients 2, 3, 5. Previous molecular biology studies have also shown that glioblastoma may progress through multiple signaling pathways 13, 14. Exosomes and their contents play an important role in glioblastoma progression and therapeutic resistance while ionizing radiation also played a positive role in the secretion of exosomes 24-26.

In this study, exosomes induced by low-dose radiation (ldrEXOs) were identified as an important factor that impacted glioblastoma progression and radioresistance. ldrEXOs promoted the expression of γ-H2AX which has a key role in DNA double-strand break repair in glioblastoma radioresistance 32. This result was similar to that of previous research by Qiupeng Zheng et al 22. They noted that exosomes derived from AHIF-overexpressing glioblastoma cells promoted viability, invasion, and radioresistance 22. However, the study of Farias et al. demonstrated that some sorts of exosomes derived from special sources can enhance radiotherapy-induced cell death in malignant tumors 28. And the regulatory function of exosomes derived from mesenchymal stem cells is quite different from that of ldrEXOs 28.

Moreover, the content of exosomes induced by radiation needs to be further studied, which is one of the reasons why the function of radiation-induced exosomes is controversial 15. This study identified for the first time that ldrEXOs are enriched with circRNAs and high-dose radiation did not significantly promote the secretion of exosomal circRNAs. It implied that the regulatory function of ldrEXOs in glioblastoma may depend on exosomal circRNAs. In the present study, ldrEXOs-derived circ-METRN played a key role in the tumor progression and radioresistance while ldrEXOs with knockdown of circ-METRN did not exhibit a similar tumor-promoting function. This effect of exosomal circ-METRN is similar to that in the research of Ding et al 23. In their research, exosomal circNFIX was up-regulated in the serum of TMZ-resistant cases 23. Exosomal circNFIX from TMZ-resistant cells conferred TMZ resistance to recipient sensitive cells through promoting cell migration and invasion and suppressing cell apoptosis under TMZ exposure 23. Han et al. also found that Circ-HIPK3 was increased in TMZ-resistant malignant glioma cells and their exosomes 33. Furthermore, circRNA has been identified as a “miRNA sponge” that functions as a miRNA inhibitor 14. This circRNA-miRNA regulatory network affects target genes and ultimately regulates malignant progression 14. In other malignant tumors, the tumor-promoting effects of exosomal circRNAs can be observed. Exosomal circRNA PDE8A promotes pancreatic cancer invasion via the miR-338/MACC1/MET pathway 16. Exosomal circRNA-100338 enhances invasiveness, metastasis, and angiogenesis in hepatocellular carcinoma 17. Gastric cancer-derived exosomal circRNA ciRS-133 promotes white adipose browning by targeting the miR-133/PRDM16 pathway 18. Exosomal circPACRGL promotes colorectal cancer progression via the miR-142-3p/miR-506-3p/TGF-β1 axis 19. Exosome circ_0044516 promotes prostate cancer cell proliferation and metastasis as a potential biomarker via miR-29a-3p 21. In the present study, this sponging relationship was confirmed between circ-METRN and miR-4709-3p. This circ-METRN-miR-4709-3p network had a significant regulatory effect on the progression of glioblastoma, which is consistent with the results of previous studies 14. The promoting effect of ldrEXOs on glioblastoma progression and radioresistance can be decreased by treatments of miR-4709-3p mimics, suggesting that miR-4709-3p may play a tumor-suppressing role in glioblastoma. Therefore, the regulatory function of ldrEXOs in glioblastoma may depend on exosomal circ-METRN, and exosomal circ-METRN exerts its regulatory functions in promoting glioblastoma progression and radioresistance through sponging miR-4709-3p.

Besides, GRB14 was determined as a direct target of miR-4709-3p, and miR-4709-3p plays a further role in glioblastoma cell inhibition by targeting GRB14. In this study, increasing the expression level of GRB14 can rescue the proliferation, migration, and invasion of glioblastoma cells, despite the different expression levels of miR-4709-3p in different groups. This coincides with the previous studies that GRB14 could promote tumor progression 34, 35. And GRB14 is highly expressed in malignant tumor cells and was regarded as a predictor of poor prognosis in tumor patients 34, 35. However, the expression level, role, and prognostic value of GRB14 in malignant tumors had often been controversial 34-37. Some other studies suggested that GRB14 was down-regulated in malignant tumor cells and can be used as a marker for good prognosis in cancer patients 36, 37. Detailed analysis of these previous studies revealed that the controvery may origin in the different binding pattern of GRB14 domains to growth factor receptors 38. In fact, the functional GRB14 binded to different growth factors with its various domains 38. For instance, GRB14 can bind to some growth factor receptors, including PDGFR, through its SH2 domain 38. Differently, GRB14 can bind to insulin receptors mainly through small domains (BPS or PIR domains) adjacent to the SH2 domain 38. Thus, GRB14 can bind to different growth factor receptors through different domains, and different binding patterns may lead to different effects 38.

The mechanism that GRB14 promoted tumor progression may be related to the downstream signal transduction pathway PDGFR. In cells, GRB14 can form a fusion protein with glutathione S-transferase, which had a strong binding ability with PDGFR, while the expression of GRB14 is not affected by the binding mode of PDGFR-GRB14 39. In this present study, the expression of PDGFRα was impacted by GRB14. Inhibition of GRB14 attenuated the tumor-promoting function of ldrEXOs although circ-METRN was highly expressed in glioblastoma cell lines previously treated with ldrEXOs. Up-regulating PDGFRα can recover the tumor-promoting function of ldrEXOs in glioblastoma cell lines previously treated with inhibition of GRB14. These results indicated that PDGFRα may be located downstream of the signal transduction pathway and may also underscore a critical role in glioblastoma progression and radioresistance. This is consistent with previous studies on the carcinogenic effects of PDGFR 40-43. H Feng et al. also determined that PDGFRα can promote the progression of glioblastoma by regulating the expression of Akt, Erk1/2, and other genes in the glioblastoma-related signal transduction pathway 40. Similar results were found in our previous research 40. Taken together, low-dose radiation-induced exosomal circ-METRN may exert its positive regulatory functions in glioblastoma progression and radioresistance via miR-4709-3p/GRB14/PDGFRα pathway.

We further explored the clinical value of low-dose radiation-induced exosomal circ-METRN (detected in the early stage of radiotherapy). This study provides strong evidence for the identification of early and rapid recurrence of glioblastoma. In the early stage of radiotherapy, low-dose radiation may promote the release and entry of exosomes into the bloodstream, thus affecting the progression and radioresistance of glioblastoma, and finally leading to the recurrence. This tumor-promoting effect of low-dose radiation-induced exosomal circ-METRN enables it to be an effective biomarker and a therapeutic target for glioblastoma patients with poor prognosis. Meanwhile, serum exosomal circ-METRN can also assist MRI diagnosis in the early detection of recurrence in glioblastoma patients. In the very early progression, the specificity of MRI diagnosis is limited because the postoperative inflammation, brain edema, and pseudoprogression can all appear as slight abnormal signals on MRI 4, 6. Our study revealed a novel diagnosis method that combined MRI and serum exosomal circ-METRN levels detected in the early stage of radiotherapy. This combined diagnosis method of abnormal signals improves the accuracy of MRI diagnosis in detecting the very early recurrence of glioblastoma. Therefore, low-dose radiation-induced exosomal circ-METRN (detected in the early stage of radiotherapy) has important clinical values. For patients who display high levels of exosomal circ-METRN (detected in the first week of radiotherapy) or who suffer a very early recurrence, it may be essential to seek other more effective and aggressive individualized treatments.

In summary, the present study is the first to examine the circ-METRN levels in low-dose radiation-induced exosomes and to reveal the role of exosomal circ-METRN via miR-4709-3p/GRB14/PDGFRα pathway in understanding the pathogenesis of glioblastoma, providing novel insights for identifying new biomarkers or potential theranostic targets.

## Supplementary Material

Supplementary tables.

## Figures and Tables

**Table 1:** Clinical characteristics of glioblastoma patients at baseline

Variable	Characteristics					
	circ-METRM in glioblastoma tissue			circ-METRN in SE (first week of radiotherapy)		
	Low (<8.499)	High (≥8.499)	*P* value	Low (<2.560)	High (≥2.560)	*P* value
**Age**						
<50	19	22	0.988	20	21	0.525
≥50	20	23		24	19	
**Gender**						
Male	19	20	0.700	18	21	0.293
Female	20	25		26	19	
**Performance status score (ECOG)**						
0-2	22	27	0.743	25	24	0.771
>2	17	18		19	16	
**Location of the tumor**						
Right	9	14	0.188	13	10	0.307
Left	15	20		20	15	
Median and/or bilateral	15	11		11	15	
**Multifocal tumor**						
Yes	4	3	0.558	5	2	0.298
No	35	42		39	38	
**Signs and symptoms**						
Epilepsy	10	5	0.292	10	5	0.016
Headache	4	8		10	2	
High intracranial pressure	4	8		6	6	
Mental status disorders	11	7		7	11	
Sensory-motor deicit	5	8		6	7	
Other	5	9		5	9	
**Tumor molecular profile**						
IDH wild type	36	42	0.858	38	40	0.015
IDH mutated type	3	3		6	0	
MGMT methylated	5	4	0.567	6	3	0.370
MGMT unmethylated	34	41		38	37	
**Postoperative therapy**						
Radiotherapy	30	35	0.876	31	34	0.101
Radiotherapy+systematic treatments	9	10		13	6	
**Abnormal MRI signals during radiotherapy**						
Yes	15	24	0.136	17	22	0.177
No	24	21		27	18	

SE: serum exosome; ECOG: Eastern Cooperative Oncology Group; MGMT: methylguanine methyltransferase; IDH: isocitrate dehydrogenase; SD: standard deviation; MRI: Magnetic resonance imaging.

## Abbreviations

ldrEXOs: low-dose radiation-induced exosome
LDR: low-dose radiation
HDR: high-dose radiation
ldrEXOs_siCIRC: exosome derived from cells treated with knockdown of circ-METRN
circ-METRN: the circular METRN RNA
linear-METRN: the linear METRN mRNA
circRNAs: circular RNAs
miRNAs: microRNAs
METRN: meteorin, glial cell differentiation regulator
PDGFRα: Platelet-derived growth factor receptor alpha
GRB: growth factor receptor-bound protein
PNBT: peritumoral normal brain tissue
NC: negative control
NHA: normal human astrocytes
DAPI: 4, 6-diamidino-2-phenylindole
siRNA: small interfering RNA
FISH: fluorescence *in situ* hybridization
RT-PCR: quantitative real-time PCR
MTT: 3-(4,5-dimethyl-2-thiazolyl)-2,5-diphenyl-2-H-tetrazolium bromide
HEK: human embryonic kidney
SD: standard deviation
OS: overall survival
DFS: disease-free survival
