# Supplementary material for: Identification of low-dose radiation-induced exosomal circ-METRN and miR-4709-3p/GRB14/PDGFRα pathway as a key regulatory mechanism in Glioblastoma progression and radioresistance: Functional validation and clinical theranostic significance
Source: Int J Biol Sci. 2021 Mar 2;17(4):1061–78. doi: 10.7150/ijbs.57168 (PMC8040305; doi:10.7150/ijbs.57168)
Supplement: Supplementary file 1 — Supplementary tables. [file ijbsv17p1061s1.pdf]

Table S1. Primers used for RT-PCR

Gene/RNA	Forward primers (5'→3')	Reverse primers (5'→3')
linear METRN mRNA	CATGCCTCCCCACAGTAATTC	AAGCTTGCATGAGTGCAGGT
GRB14	CTGTGGGGGTTCAGACTCAC	AAGCTGTGAGCATTCTGGCT
PDGFRα	AGAGAGGGGACCCAGAACAA	ACTGCGTGTGTTTTTGGCAG
GAPDH	TGACCTTTCTGTAGCTGGGG	CAAGCCCACCCCTTCTCTAA
circ-METRN	TGCTTTGGAGGTGATGGGAC	GGGTACAGCCACTCAACCG
miR-4709-3p	GAAGAGGAGGTGCTCTGT	GGTCCAGTTTTTTTTTTTTTTTGCT
U6	CTCGCTTCGGCAGCACA	AACGCTTCACGAATTTGCGT

Table S2. Small interfering RNAs (siRNAs) involved in this study

Gene/RNA	siRNA (5'→3')	siRNA scrambled (5'→3')
linear METRN mRNA	GGAUGCAGAUGCCUGAUGAGU	GGGUCAGAGCUGUCGUUAGAA
GRB14	GGGAGAAUUAGAACUAGAUGA	GUUGGAAGAUACGUGAAAGAA
PDGFRα	GCUUAAUUGCUGAUACCAUAUG	GCAGAUAUAUUACUUGUCCUAA
circ-METRN	GGCAAGCAGAUCAUGAGGUCA	GAGGUGACUCCAACGGAUAGA

Table S3. The predicted molecular weights, antibodies with vendor and dilutions

Proteins	molecular weights	vendor	dilutions
CD63	26 kDa	ThermoFisher Scientific	1:1000 (WB)
TSG101	47kDa	ThermoFisher Scientific	1:1000 (WB)
γ-H2AX	15kDa	ThermoFisher Scientific	1:2000 (WB)
GAPDH	36 kDa	Santa Cruz Biotechnology	1:1000 (WB)
GRB14	58 kDa	ThermoFisher Scientific	1:1000 (WB)
PDGFRα	130 kDa	ThermoFisher Scientific	1:1000 (WB)
PI3K	85 kDa	Santa Cruz Biotechnology	1:1000 (WB)
AKT	60 kDa	Santa Cruz Biotechnology	1:1000 (WB)
ERK	44 kDa	Abnova	1:2000 (WB)
MEK1/2	45 kDa	Abnova	1:2000 (WB)

WB western blot

Table S4. Inclusion criteria

Inclusion criteria	Details
General information available	Newly diagnosed glioblastoma (WHO grade IV), Eastern Cooperative Oncology Group (ECOG) performance status score, normal liver, kidney and bone marrow functions.
Operation information available	Gross total resection was confirmed by pathological and imageological diagnoses.
Radiotherapy information available	Radiotherapy was performed within 4-6 weeks after surgery using volumetric modulated arc therapy; the dose prescribed: 54-60Gy with a daily fraction of 1.8-2Gy (5 fractions per week); the target delineation performed according to international guidelines.
Tumor molecular profile available	IDH type and MGMT methylated information
Specimens available	Fresh frozen pathological specimens and serum specimens were available.
Available information on systemic treatment	Temozolomide was administered as following: orally, once daily, at 75 mg/m^2, starting on the first day of radiotherapy and continuing for the whole treatment; after a 4-week break, administered at 150-200 mg/m^2 orally, once daily, for 5 consecutive days every 28 days up to 12 cycles, or until disease progression.

Table S5. Exclusion criteria

Exclusion criteria	Details
General information unavailable	Detailed information about the diagnosis, performance status, liver, kidney and bone marrow functions was unavailable.
Operation	Operation information was unavailable. Patients underwent incomplete resection.
Radiotherapy	Radiotherapy information was unavailable. Patients underwent nonstandard or palliative radiotherapy.
Tumor molecular profile unavailable	IDH type or/and MGMT methylated information was unavailable.
Specimens unavailable	Fresh frozen pathological specimens or/and serum specimens were unavailable.
Systemic treatment information unavailable	Patients underwent nonstandard systemic treatments.

Table S6. Univariate and multivariate analyses of factors associated with OS and DFS

Variable	Univariate for OS, P value	Multivariate for OS			Univariate for DFS, P value	Multivariate		
		HR	95% CI	P value		HR	95% CI	P value
Age	0.927	NA	NA	NA	0.411	NA	NA	NA
Gender	0.533	NA	NA	NA	0.011	1.884	1.161-3.058	0.010
Performance status score	0.090	1.632	1.010-2.637	0.045	0.408	NA	NA	NA
Location of the tumor	0.405	NA	NA	NA	0.942	NA	NA	NA
Multifocal tumor	0.896	NA	NA	NA	0.374	NA	NA	NA
Signs and symptoms	0.002	0.874	0.765-0.998	0.047	0.479	NA	NA	NA
IDH type	0.087	NA	NA	NA	0.840	NA	NA	NA
MGMT methylated/ unmethylated	0.095	NA	NA	NA	0.473	NA	NA	NA
Postoperative Therapy	<0.0001	0.223	0.117-0.426	<0.0001	0.188			<0.0001
circ-METRN levels								
Glioblastoma tissues	<0.0001	1.516	1.301-1.766	<0.0001	<0.0001	1.273	1.076-1.505	0.005
SE (before operatation)	0.013	NA	NA	NA	0.756	NA	NA	NA
SE (before radiotherapy)	0.096	NA	NA	NA	0.033	NA	NA	NA
SE (first week of radiotherapy)	0.003	1.325	1.089-1.612	0.005	<0.0001	3.309	2.303-4.754	<0.0001
SE (after radiotherapy)	0.173	NA	NA	NA	0.127	NA	NA	NA
Abnormal MRI signals during radiotherapy	0.667	NA	NA	NA	0.218	NA	NA	NA

OS: overall survival; DFS: disease-free survival; SE: Serum exosome; MGMT: methylguanine methyltransferase; IDH: isocitrate dehydrogenase; MRI: Magnetic resonance imaging; HR: hazard ratio; CI: confidence interval; NA: not adopted

Table S7. Combined diagnosis of abnormal MRI signals during radiotherapy

Diagnosis methods		Pathological diagnosis on the location of abnormal MRI signal		Total
		Positive	Negative	
Combined diagnosis	Positive (recurrence)	21	1	22
	Negative	3	24	27
MRI	Positive	4	23	27
	Negative/undiagnosed	20	2	22
Total		24	25	49

MRI: magnetic resonance imaging
